# Olfactory deficits in aging and Alzheimer’s—spotlight on inhibitory interneurons

**DOI:** 10.3389/fnins.2024.1503069

**Published:** 2024-12-16

**Authors:** Kaoutar Elhabbari, Siran Sireci, Markus Rothermel, Daniela Brunert

**Affiliations:** Institute of Physiology, RG Neurophysiology and Optogenetics, Medical Faculty, Otto-von-Guericke-University, Magdeburg, Germany

**Keywords:** olfaction, interneurons, aging, Alzheimer’s disease, inhibition

## Abstract

Cognitive function in healthy aging and neurodegenerative diseases like Alzheimer’s disease (AD) correlates to olfactory performance. Aging and disease progression both show marked olfactory deficits in humans and rodents. As a clear understanding of what causes olfactory deficits is still missing, research on this topic is paramount to diagnostics and early intervention therapy. A recent development of this research is focusing on GABAergic interneurons. Both aging and AD show a change in excitation/inhibition balance, indicating reduced inhibitory network functions. In the olfactory system, inhibition has an especially prominent role in processing information, as the olfactory bulb (OB), the first relay station of olfactory information in the brain, contains an unusually high number of inhibitory interneurons. This review summarizes the current knowledge on inhibitory interneurons at the level of the OB and the primary olfactory cortices to gain an overview of how these neurons might influence olfactory behavior. We also compare changes in interneuron composition in different olfactory brain areas between healthy aging and AD as the most common neurodegenerative disease. We find that pathophysiological changes in olfactory areas mirror findings from hippocampal and cortical regions that describe a marked cell loss for GABAergic interneurons in AD but not aging. Rather than differences in brain areas, differences in vulnerability were shown for different interneuron populations through all olfactory regions, with somatostatin-positive cells most strongly affected.

## Introduction

Maintaining cognitive function is a central point in preserving the quality of life in aging individuals (Majmundar and Hayward, 2018). Both healthy aging as well as dementia-inducing “pathological” aging show, to differing degrees, signs of cognitive decline in the form of memory loss, as well as a decrease in attention and executive function (Harada et al., 2013). Alzheimer’s disease (AD) is a critical public health issue and the leading cause of dementia, with an estimated 32 million persons suffering from AD dementia globally (Gustavsson et al., 2023), constituting between 10 and 30% of individuals above the age of 65 (Masters et al., 2015; Prince et al., 2015). AD is characterized by the buildup of two proteins: Amyloid beta (Aβ), which accumulates extracellularly as plaques, and hyperphosphorylated *τ*, which accumulates intracellularly as neurofibrillary tangles (Braak and Braak, 1991). These neuropathological changes are detectable in olfactory areas in the earliest stages of AD, even preceding damage to the entorhinal cortex (Kovács et al., 2001).

Besides a decrease in cognitive capabilities, olfactory perceptual performance decline is also a widespread occurrence (Kondo et al., 2020; Tzeng et al., 2021). This decline in olfactory function is associated with various measures of cognition and memory performance decline (Wilson et al., 2007; Devanand, 2016; Olofsson et al., 2016; Papadatos and Phillips, 2023). The severity of olfactory dysfunction has been associated with the rate of cognitive decline in patients with dementia (Dintica et al., 2019) showed that odor identification impairment has an even higher predictive value for cognitive decline than deficits in verbal episodic memory. Most often, olfactory deficits are noticeable years before the first signs of cognitive decline are apparent (Swan and Carmelli, 2002; Wilson and Mainen, 2006). As this line of research is of high value for early diagnostics of AD, many publications have investigated the correlation of aging and disease severity with olfactory symptoms, and this topic has been the subject of many reviews (Attems et al., 2014; Doty and Kamath, 2014; Mobley et al., 2014; Franks et al., 2015; Murphy, 2019; Kondo et al., 2020; Olofsson et al., 2021). Therefore, we would like to give a brief overview:

Olfactory perceptual decline is highly prevalent in aging individuals, with smell loss experienced by around half the population between 65 and 80 years of age and about three-quarters of the population by age 80 (Doty and Kamath, 2014). Consequences of this loss in olfactory abilities are dietary changes, i.e., anorexia due to reduced pleasantness of food or obesity, reduced threat detection for environmental hazards or spoiled food, and problems in personal hygiene (Olofsson et al., 2021). Additionally, it has been shown that olfactory assessment can identify individuals with a higher risk of developing dementia (Graves et al., 1999; Devanand et al., 2008; Conti et al., 2013; Tebrügge et al., 2018; Olofsson et al., 2020). Despite this high relevance of olfactory decline in aging individuals, the characterization of “normal” olfactory aging is far from complete. It is generally assumed that two major contributing factors to changes in olfactory perception are a reduction in the number of olfactory receptor neurons and a more than 50% reduction in the number of adult-born periglomerular cells within the olfactory bulb due to reduced stem cell proliferation in the subventricular zone (SVZ) (Mobley et al., 2013; Mobley et al., 2014). These changes in more peripheral olfactory areas, however, cannot fully account for the nature of modifications in olfactory perception, which are more associated with odor identification and odor memory than detection thresholds (Cerf-Ducastel and Murphy, 2009; Sela et al., 2009).

In AD, similar changes in odor identification as well as odorant detection and discrimination have been shown (Dan et al., 2021), which point to defects, especially in central regions, as being responsible for olfactory dysfunction (Rawson, 2006). Despite the apparent association between olfactory and cognitive performance, the underlying causes of olfactory deficits in aging and AD are unclear.

Compared to cognitive changes, systematic research on the cellular nature of olfactory dysfunctions is in its infancy. The hippocampus (HC) has been shown to undergo many structural changes in aging and AD, ranging from astrogliosis, microgliosis, changes in mitochondria morphology, and reduction in neurogenesis to cell morphology changes, synaptic and cellular decline, and decrease in volume (Hullinger and Puglielli, 2017). Besides these well-established changes, the hippocampus, as well as cortical structures, have been shown to exhibit hyperexcitability manifesting in increased network activity, epileptic activity, slowing of neural oscillations, and reductions in waveform complexity (Kazim et al., 2021; Maestú et al., 2021; Tok et al., 2022).

Changes in the intrinsic properties of excitatory neurons or changes in inhibitory drive could cause these hyperexcitable states. As GABAergic interneurons were initially shown to be resistant to Aβ toxicity (Pike and Cotman, 1993) much research in AD has focused on excitatory neurons in different brain areas. Recent research, however, has strongly implicated a reduction of inhibitory input to local networks (Xu et al., 2020; Tok et al., 2022) that could either originate in a reduction of long-range inputs or a decrease in local inhibitory tone. Local inhibitory interneurons, here, are the most promising avenue of research as they account for 75% of the total inhibitory input hippocampal neurons receive (Mody and Pearce, 2004). Detectable changes in GABAergic interneurons are a reduction of function (Verret et al., 2012) but predominantly a loss of neuronal numbers in hippocampal and cortical regions of both AD patients as well as mouse models (Xu et al., 2020).

Likewise, aberrant neuronal network activity has been identified as one of the hallmarks of the aging brain (Mattson and Arumugam, 2018). This line of research presents the possibility of connecting cellular changes to behavioral dysfunctions (Maestú et al., 2021). Despite reports on interneuron loss (Cha et al., 1997; Potier et al., 2006) the predominant cause for aberrant excitation seems to be a reduction of inhibitory synapses and GABAergic transmission (Rozycka and Liguz-Lecznar, 2017). GABAergic interneurons and their influence on local networks are, therefore, believed to play a large part in the occurrence of cognitive deficits and neuropathology in both AD (Xu et al., 2020) and aging (Rozycka and Liguz-Lecznar, 2017).

**Figure 1:**
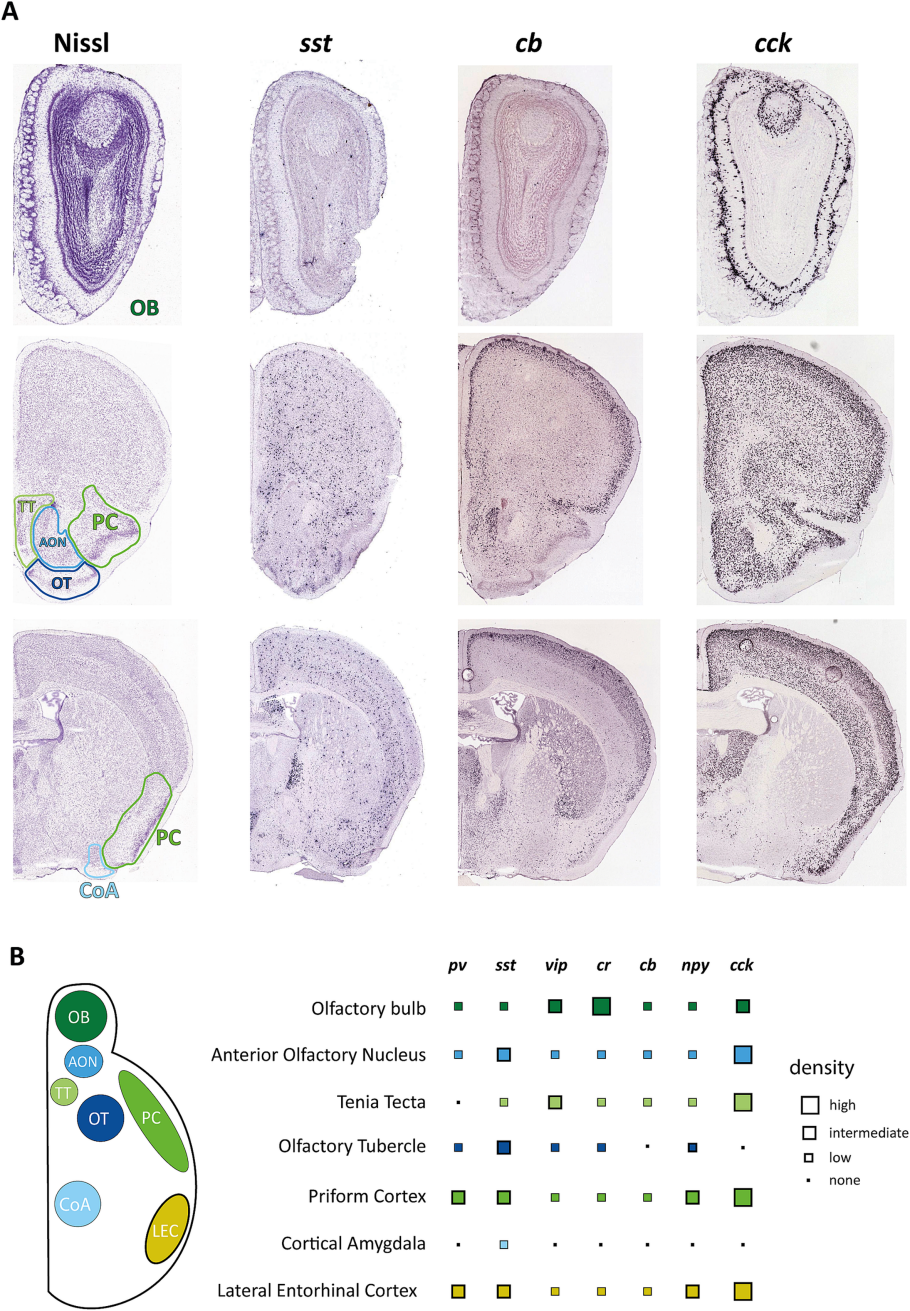
Distribution of interneuron markers in primary and secondary olfactory areas. **(A)** Example pictures from the Allen Mouse Brain Atlas showing coronal brain sections containing olfactory areas in reference to Nissl staining of these regions. Images were derived from Allen Mouse Brain Atlas and Allen Reference Atlas, Experiment 77869074 (https://mouse.brain-map.org/experiment/show/77869074) for cholecystokinin (*cck*), Experiment 1001 (https://mouse.brain-map.org/experiment/show/1001) for somatostatin (*sst*), and Experiment 71717640 (https://mouse.brain-map.org/experiment/show/71717640) for calbindin (*calb1*) (Allen Institute for Brain Science, 2004; Lein et al., 2007). **(B)** Overview on the distribution of the most common interneuron markers. Data points represent estimates from ISH experiments from the Allen Mouse Brain Atlas but have been matched to reports of protein expression as far as these data are available. *pv*, parvalbumin; *sst*, somatostatin; *vip*, vasoactive intestinal peptide; *cr*, calretinin; *cb*, calbindin; *npy*, neuropeptide y; *cck*, cholecystokinin.

**Figure 2:**
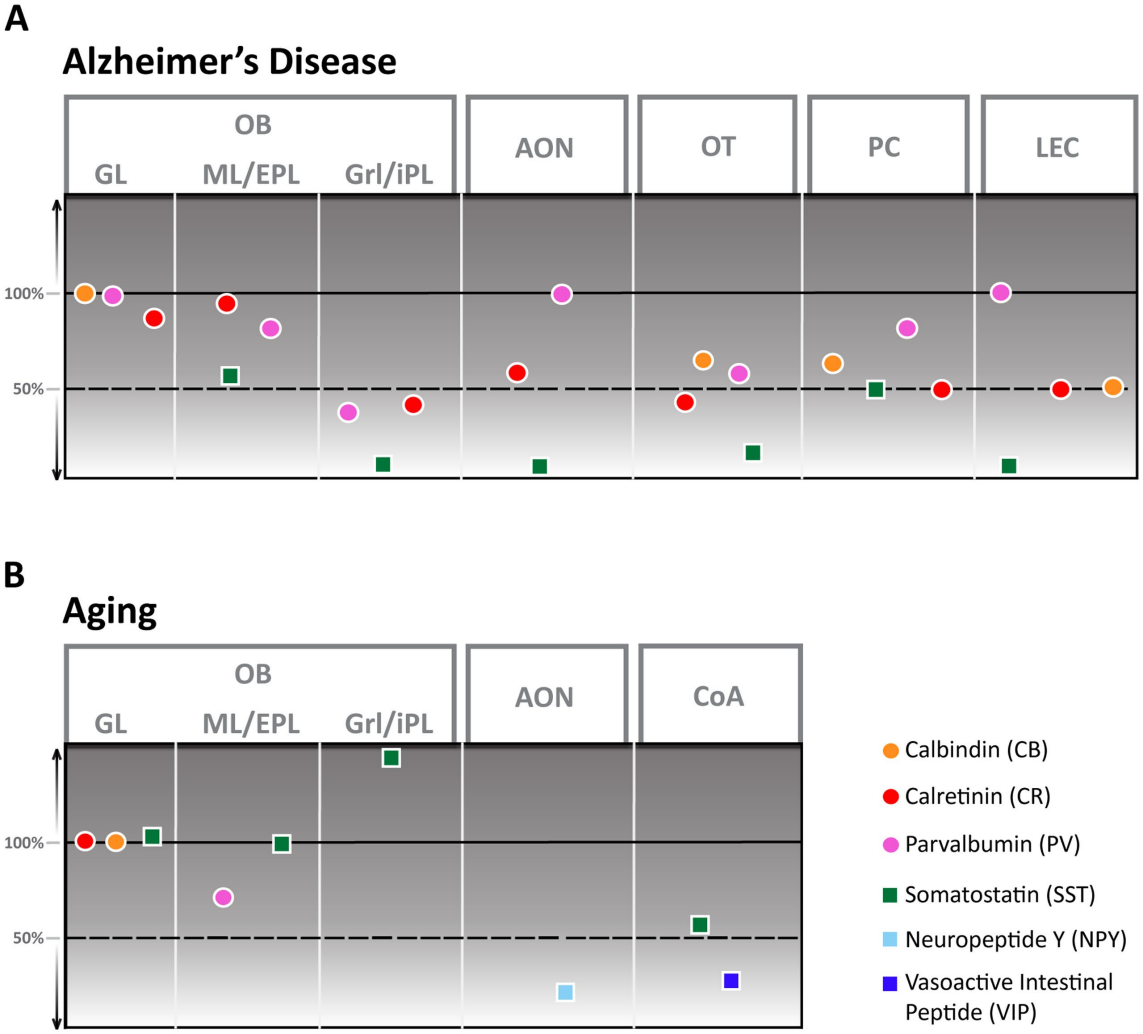
Alzheimer’s Disease **(A)** and aging **(B)** derived changes of interneuron numbers in different olfactory areas. AON, anterior olfactory nucleus; CoA, cortical amygdala; EPL, external plexiform layer; GL, glomerular layer; Grl, granule cell layer; iPL, internal plexiform layer; ML, mitral cell layer; OB, Olfactory bulb; OT, olfactory tubercle; PC, piriform cortex; LEC, lateral entorhinal cortex.

Olfactory sensory circuits, so far, have rarely been probed for changes in excitability, although hyperexcitability in the olfactory bulb was shown to lead to impaired olfactory behavior in a mouse model for Fragile X syndrome (Kuruppath et al., 2023). However, several reports exist of reduced numbers of inhibitory interneurons in several olfactory areas. As the underlying cause of olfactory decline in AD and aging is still undefined, we felt it was time to spotlight the interneuron populations of the OB and olfactory cortex regions. We will give an overview of the interneuron populations described so far (Figure 1). This we take as a basis to outline changes to these interneuron populations in AD and aging (Figure 2).

## Interneuron composition of the primary olfactory cortex and changes in healthy and pathological aging

### Common interneuron markers

Inhibitory interneurons of different brain areas can be incredibly diverse, with each brain area featuring different populations with unique properties. Inhibitory interneurons, in general, are defined by various morphological, molecular, and physiological features, including their location, morphology, spiking properties, connections, and expression profile (Kepecs and Fishell, 2014). The easiest way to broadly classify these neuronal subtypes is through their expression of several specific interneuron markers. The description of interneuron populations through these markers has become more popular as they provide a means to label particular populations with optogenetic tools. According to the Petilla terminology of molecular classification of cortical interneurons (Ascoli et al., 2008), these markers are the calcium-binding protein parvalbumin (PV) and the neuropeptides somatostatin (SST), vasoactive intestinal peptide (VIP), neuropeptide Y (NPY) and cholecystokinin (CCK) (DeFelipe et al., 2013) with further subclassification with other calcium-binding proteins like calretinin (CR) and calbindin (CB) (DeFelipe et al., 2013). Of these, PV and SST neurons make up the majority of interneurons of the neocortex, but different areas have been shown to exhibit varying ratios of interneuron markers (Whissell et al., 2015).

We have gathered information on interneuron subtypes of different olfactory areas and listed them concerning location, morphology, and function, as far as this information is available. We have also gathered information on their changes in AD and aging to provide a starting point for discussing the possible involvement of interneurons in the etiology of olfactory dysfunction.

### Olfactory bulb (OB)

The OB is the brain’s first relay station of olfactory information. Olfactory receptor neurons in the olfactory epithelium detect odor molecules and send unbranched axons to the OB (Klenoff and Greer, 1998). There, they converge and form synaptic structures called glomeruli with output neurons, tufted and mitral cells, which are located in the external plexiform layer (EPL) and mitral cell layer (ML), respectively (Nagayama et al., 2004; Sarafoleanu et al., 2009).

The OB is unique as it contains a much higher proportion of interneurons in relation to excitatory neurons than other brain areas [100:1 compared to 1:5 (Kim and Choe, 2020)] allowing for robust processing and modulation of olfactory information at this early processing stage (Wachowiak and Shipley, 2006; Brunert and Rothermel, 2021). Furthermore, it receives adult-born neuroblasts from the subventricular zone (SVZ) through the rostral migratory stream. These neuroblasts get integrated into the glomerular or granule cell layer and constitute a separate population of predominantly CR-positive interneurons (Kim and Choe, 2020). The OB exhibits a heterogeneous population of GABAergic interneurons that can be differentiated by their morphology, electrophysiological properties, and connectivity patterns (Markram et al., 2004) but are described mainly by their soma location (Nagayama et al., 2014) as periglomerular cells (PGCs) (also juxtaglomerular cells), granule cells, or interneurons of the mitral and internal plexiform layer. Though PGCs constitute only about 4% of OB interneurons, they have been well described, especially by their expression profile (Kosaka and Kosaka, 2005) and synaptic processing (Wachowiak and Shipley, 2006). They are characterized mainly by three different molecular markers: CB, CR, and tyrosine hydroxylase (TH). The latter constitutes a population of approximately 10% of PGCs, also called short axon cells, that are GABAergic and dopaminergic in nature and connect multiple glomeruli. Other PGCs are connected to a single glomerulus, express CB or CR, and about 30% of them receive monosynaptic input from olfactory receptor neurons (Kiyokage et al., 2010). Functions of PGC have been shown in presynaptic inhibition of OB input (Pírez and Wachowiak, 2008) as well as top-down modulation of OB circuits (De Saint Jan, 2022). Neurons in the external plexiform layer, deeper in the OB, only constitute 2% of the neurons and are characterized by the expression of PV or corticotropin-releasing hormone (CRH). PV-expressing interneurons are typically axonless and have electrophysiological properties similar to fast-spiking PV cells found throughout the cortex (Kato et al., 2013). They furthermore are strongly connected to mitral cells via dendrodendritic synapses and have been reported to modulate the gain of olfactory bulb output (Kato et al., 2013). Another type of interneuron expressing trophoblast glycoprotein gene, 5T4, was found to be located primarily in the mitral cell layer but is low in number (Yoshihara et al., 2014) and has been shown to play a role in olfactory detection and discrimination.

Granule cells (GC) of the granule cell layer are the most abundant type of interneurons in the OB. These neurons are axonless, CR-expressing cells that can be divided into three subtypes based on morphology, connectivity, and expression (Lledo et al., 2008). They provide strong dendrodendritic inhibition to either tufted cells (superficial GCs) or mitral cells (deep GCs), thus providing the cellular basis for increasing sensory resolution and discrimination through lateral inhibition (Egger and Kuner, 2021).

Expression of other typical interneuron markers is distributed within these already described groups. Like the relatively sparse expression of PV, SST expression is also lower in the OB compared to neocortical areas. In the OB, SST is predominantly expressed in CR-positive neurons of the EPL as well as in a subtype of short axon cells in the granule cell layer (Lepousez et al., 2010) but ablation of SST receptors significantly affects olfactory discrimination (Nocera et al., 2019). Information is also available on VIP-positive interneurons located primarily in the glomerular layer. Knocking out this population of neurons also causes deficiencies in odor detection and discrimination (Wang et al., 2022).

Aging in humans has been shown to elicit a substantial reduction in the volume of all cellular layers, most notably the glomerular layer (Doty and Kamath, 2014). Despite this apparent change in the human OB, the exact nature of these age-related changes on a cellular level is still largely unclear since aging rodents seem to either show no (Richard et al., 2010) or little (Hwang et al., 2004) change in cellular composition. While numbers of CB and CR neurons stay constant (Mobley et al., 2013), an aging-associated decline in cell numbers has only been shown for PV-interneurons. These decrease by about 30% in rats between the ages of 12 and 24 months (Hwang et al., 2003) while the amount of SST-positive cells increases strongly, however, exclusively in the granule cell layer (Figure 2B; Hwang et al., 2004). In contrast, it was shown that synaptic density in the glomerular layer strongly decreases in aging mice (Richard et al., 2010) with potential effects on olfactory discrimination.

In AD, the OB shows a high number of senile plaques and strong tauopathy in the early stages of the disease (Kovács et al., 2001). Cellular changes in the OB of an AD mouse model are pronounced (Saiz-Sanchez et al., 2013; La Rosa-Prieto et al., 2016): Though CB-positive neurons were not altered in number, CR-positive neurons showed a significant reduction in the granule cell layer compared to healthy control mice, at least in younger animals. PV cell numbers were lower in younger animals of an AD mouse model than controls but higher in animals older than a year, thus showing a solid age-dependent modulation. SST-positive neurons, in contrast, showed a substantial decrease, with the strongest reduction occurring in the granule cell layer. At least this substantial reduction in SST-positive cells seems to be present also in AD patients (Figure 2A; Saiz-Sanchez et al., 2020). How these changes would affect olfactory behavior is still unclear.

### Anterior olfactory nucleus (AON)

The AON is located in the olfactory stalk between the OB and the piriform cortex. Though the AON receives the predominant amount of olfactory information input from the olfactory bulb (Chae et al., 2022) and is intensely connected to olfactory and non-olfactory areas (Brunjes et al., 2005; Brunert et al., 2023), its role for olfactory perception is still unclear. So far, it has been shown that it can store olfactory contextual memory (Aqrabawi and Kim, 2020) can intensely regulate information output from the olfactory bulb (Medinaceli Quintela et al., 2020), and might have a role in social recognition (Oettl et al., 2016). However, a clear picture has still to emerge. Likewise, the interneurons of the AON have not been tied to a clear behavioral output.

The AON itself is a two-layered structure consisting of a pars principalis (pP) and a pars externa (pE) structure, with pP composed of four different subsections that differ in connectivity and, potentially, function. pP and pE are very different in their composition, though both exhibit a surprising variety of different interneurons (Kay and Brunjes, 2014) for such simplified cortical structures. Just a limited characterization of glutamate dehydrogenase 1 positive cells in pP based on their electrophysiology and morphology found at least five possible distinct subtypes, while immunohistochemical characterization showed 13 possible subtypes (Kay and Brunjes, 2014). This histochemical characterization showed an overlapping but distinct marker composition compared to the hippocampus or neocortex, with CB, CR, and PV as the most prominent markers. VIP and SST-positive neurons could also be found in significant numbers, while the number of NPY and CCK-positive cells was low in AON pP (Figure 1B). The number of neurons expressing one of the tested markers was higher in layer I of AON pP and could differ significantly depending on the examined subsection.

While a detailed characterization of pE interneuron composition is still pending, limited experiments have shown a high number of CR-positive cells as well as some cell types with unique properties (Kay and Brunjes, 2014).

As the olfactory bulb shows a high density of senile plaques and neurofibrillary tangles in the early phases of AD, efforts have been made to characterize the cellular changes the AON undergoes in this disease for humans and mouse models. Both human (Saiz-Sanchez et al., 2010) as well as mouse models (Saiz-Sanchez et al., 2013) show a significant reduction of SST-positive cells that manifests in mice as early as 6 months of age. In mice, the number of CR-positive cells also showed a substantial reduction in number while PV-positive cells were less vulnerable to disease progression and showed no reduction (Figure 2A; Saiz-Sanchez et al., 2013). Research on other interneuron populations in the AON during AD disease progression is still pending.

Even less is known about the changes the AON undergoes in aging; the only report comes from NPY-positive cells in the AON of rats that decline considerably in number between the ages of 12 and 24 months for all subsections of pP (Figure 2B; Hwang et al., 2001).

Despite its predominant role in olfactory processing and contextual memory, functional studies examining the association of olfactory decline in aging and AD to AON are missing so far.

### Piriform cortex (PC)

The PC is the largest cortical area receiving olfactory signals from the OB (Neville and Haberly, 2004). It consists of two parts, the anterior piriform cortex (APC) and the posterior piriform cortex (PPC), that differ strongly in connectivity (Hagiwara et al., 2012) and, most probably, function (Kadohisa and Wilson, 2006; Dhawale et al., 2010). The PC is a three-layered cortical structure that contains principal neurons in layers II and III, while interneurons are more homogenously distributed throughout all layers (Bekkers and Suzuki, 2013).

Odor processing in the PC is governed by strong feedforward as well as feedback inhibition through local recurrent networks (Franks et al., 2011; Suzuki and Bekkers, 2012). In contrast to other olfactory areas, PC has been well characterized in terms of interneuron composition (Young and Sun, 2009; Gavrilovici et al., 2010; Suzuki and Bekkers, 2010a,b; Bekkers and Suzuki, 2013; Agarwal et al., 2014). Though similar to neocortical composition, PC has a more simple makeup of inhibitory interneurons with just five main classes that can be identified in the APC (Suzuki and Bekkers, 2010a,b). There, morphologically identified neurogliaform and horizontal cells in layer I seem to provide feedforward inhibition, while bitufted, fast, and regular spiking neurons, as well as chandelier cells and deep neurogliaform cells, are located mainly in layer II and III mediating feedback inhibition (Suzuki and Bekkers, 2012). Interestingly, neither neurogliaform nor horizontal cells are positive for common interneuron markers (Suzuki and Bekkers, 2012). Neurons located in the deeper layers of APC responsible for feedback inhibition, bitufted cells, fast-spiking multipolar and regular spiking multipolar cells have been shown to stain for VIP, CB, and/or PV and SST, respectively (Suzuki and Bekkers, 2010a). Feedback inhibition is far more potent than feedforward inhibition for the PC (Franks et al., 2011) and can powerfully shape odor coding.

The PPC gets less olfactory input from the OB than the APC and more associational input, leading to the hypothesis that the APC might be responsible for odor learning and identity coding and the PPC for odor valence coding (Calu et al., 2007). Experiments suggest that the inhibitory networks in the PPC seem to be even stronger than in the APC. In the PPC, different interneuron cell types have been identified according to their firing properties (Young and Sun, 2009): Late spiking (LS) and irregular spiking (IS) cells that were predominantly located in layer I of the piriform cortex, as well as regular spiking nonpyramidal cells (RSNP) and fast-spiking (FS) neurons. All of these neurons exhibit either no (FS and LS) or minor staining for calcium-binding proteins, though low numbers of CB and CR positive neurons can be seen scattered throughout all layers, and PV cells are located in layer II (Kiselycznyk et al., 2006) Only 20% of the IS cells showed to be positive for CR, while 33% of all RSNP were positive for VIP but no calcium-binding proteins.

Multiple functions for inhibitory interneurons in PC have been shown. SST and NPY infusion to APC changed feeding behavior (Cummings et al., 1998), and PV-positive.

FS cells are the recipient of top-down dopaminergic inputs (Potts and Bekkers, 2022). Furthermore, it was shown that long-term potentiation within APC requires the inactivation of SST or PV-positive neurons by VIP-positive interneurons that act as gatekeepers for sensory processing and learning (Canto-Bustos et al., 2022).

Though APC and PPC have been hailed as the new frontier in aging research more than 10 years ago (Mobley et al., 2014) we still know little about “healthy” aging processes in the PC. There are reports that PC interneuron populations might change during the life of a mouse (Saiz-Sanchez et al., 2012), with the number of CB and PV positive neurons being higher in older mice, these data track the aging process only up until 8 months and thus do not cover the “aging” process. Reports on functional changes report cortical thickness, synaptic density, and cell numbers in layer II to remain stable in PC even in advanced ages (Curcio et al., 1985; Diamond et al., 1977) which contrasts strongly with the decline seen in the OB and olfactory epithelium (Mobley et al., 2014). One reason for this could be that the population of cortical immature neurons (cIN) of the PC is created prenatally and lays dormant inside layer II until needed (Gómez-Climent et al., 2008). The cells thus act as a lifelong neurogenic pool that reduces with age but provides new neurons during the lifespan of an animal (Ghibaudi et al., 2023).

In AD, the number of interneurons is severely affected in humans and AD mouse models. In mice, CB, CR, PV, and SST cell numbers were significantly decreased at 8 months of age (Figure 2A), with CR and especially SST showing effects in very early stages (Saiz-Sanchez et al., 2012). Here, it is interesting that PPC showed more robust effects that manifested slightly later than in the APC. In AD patients, a similar pattern in interneuron decline was shown, except that the number of PV neurons increased (Saiz-Sanchez et al., 2015). Numerous functional deficiencies of the PC in AD, including disruption of odor quality coding (Li et al., 2010), and reduction of neuromodulatory input due to noradrenergic fiber degeneration (Rajani and Yuan, 2022) or decreased excitability of pyramidal cells through activation of 2-HT2c receptors (Wang X. et al., 2023) have been shown. None of these defects have been tied to the function of inhibitory interneurons yet.

### Tenia tecta (TT)

Another system underrepresented in research on olfactory deficits is the TT. This region consists of two separate three-layered subregions, dorsal and ventral tenia tecta (dTT and vTT), which are evolutionarily derived from two different formations and present distinct cellular compositions. dTT, also called the hippocampal anterior continuation (McNamara et al., 2004), contains a more hippocampal-like cell composition (Haberly and Price, 1978) and connectivity (Brunjes et al., 2005). Reports on the presence of common interneuronal markers show a medium amount of CR-positive (Qi et al., 2022) and CB-positive neurons (Tsuneoka et al., 2017) as well as a low number of SST-positive neurons (Nocera et al., 2019). Though the dTT exhibits robust odor responses, presumed to stem from PC and lateral entorhinal cortex inputs (Cousens, 2020), the primary input of the OB is received by the vTT. This area, separated by a thin, cell-free layer from the dTT, shows less robust odor responses (Cousens, 2020) but has been shown to integrate odor information with distinct environmental and behavioral contexts of learned behaviors (Shiotani et al., 2020). Besides OB, the vTT receives inputs from the APC and PPC and medial prefrontal cortex. Projections of vTT extend to the OB, AON and APC. Cell selective studies have predominantly addressed the pyramidal cells in layers 2 and 3 of the vTT (Haberly and Price, 1978). Still, immunohistochemistry for common interneuron markers has shown the presence of these markers, calcium-binding proteins CB, CR, and PV, and neuropeptidergic cells like VIP, NPY, SST, and CCK (Brunjes et al., 2011; Bjerke et al., 2021).

Research on aging and AD has only recently included TT. Oxytocin receptor mRNA did not show any age-related changes in the rat, different from hypothalamic areas (Ravenel et al., 2024), but TT showed substantial Aβ plaque deposition and amyloid precursor protein deposition in AD mice (Tsui et al., 2022; Ono et al., 2024). Though there was no measurement of olfactory dysfunction in these studies, it has been shown that neurotoxic exposure to methylmercury leads to olfactory dysfunction while causing strong neuronal loss in the olfactory system, particularly in the vTT (Iijima et al., 2024). So far, nothing has been published on the fate of interneurons of the TT during aging or AD. Intense amyloid deposition and the close connection to hippocampal areas suggest that this area might be highly relevant to olfactory dysfunction and potentially disease progression.

### Olfactory tubercle (OT)

The OT is part of the ventral striatum and is located at the ventral part of the olfactory peduncle posterior to AON and vTT. It is a three-layered structure that, similar to PC, contains a superficial layer I that receives monosynaptic input from the OB, a cell-dense layer II, and a deeper layer 3 (Xiong and Wesson, 2016). Like other striatal regions, OT contains primarily GABAergic neurons, including local interneurons and spiny projection neurons projecting to other striatal regions and into midbrain structures (Zhang et al., 2017). It also contains the majority of the “islands of Cajella” (IC), dense clusters of GABAergic granule cells (Hsieh and Puche, 2013), scattered throughout the OT at variable locations with multiple behaviorally relevant functions for motivation and self-reward (Zhang et al., 2023). The OT displays odor-specific responses (Wesson and Wilson, 2010), is a site of multisensory integration (Wesson and Wilson, 2011), and is speculated to play a large part in coding odor valence (Gadziola et al., 2015) and the regulation of odor-guided food intake (Murata, 2020).

Similar to several other olfactory regions, different morphological types of putative interneurons have been described (Millhouse and Heimer, 1984), but these morphological types have not been matched with expression types. The OT shows expression for all of the common interneuron markers (Brunjes et al., 2011; Martin-Lopez et al., 2019; Zandt et al., 2019). Some of these show a pronounced heterogeneity in terms of localization, with CB and PV-positive neurons residing primarily in layer III while reelin-positive cells residing predominantly in layer I (Martin-Lopez et al., 2019). Direct functions for GABAergic interneurons in the OT have yet to be shown.

Aging had a measurable effect on OT, predominantly on the IC. The IC receives small numbers of adult-born neuroblasts that decline as a result of aging-related changes in the SVZ (Mobley et al., 2014). Potentially due to this process or increased accumulation of autophagosomes (Soontornniyomkij et al., 2012), the number and volume of ICs were significantly reduced in older mice (Adjei et al., 2013) with the potential to lead to depression-like behaviors (Zhang et al., 2023).

The OT of AD mouse models show a high number of senile plaques (Wesson and Wilson, 2010; Saiz-Sanchez et al., 2013) though the increase is slightly delayed compared to OB and AON in the APP/PS1 mouse line (Saiz-Sanchez et al., 2013). Multiple different types of interneurons show a decrease in numbers, like CB (Selden et al., 1994), PV, CR, and SST positive neurons (Figure 2A; Saiz-Sanchez et al., 2013). None of these interneuron populations’ functions are defined, so it remains to be determined whether and how these changes in the OT contribute to olfactory dysfunction.

### Cortical amygdala (CoA)

The CoA is, like other paleocortical areas, a three-layered structure consisting of multiple different subnuclei such as the nucleus of the lateral olfactory tract (nLOT), bed nucleus of the accessory olfactory tract (BAOT), anterior cortical amygdala posterolateral (PLCo) and posteromedial nuclei (PMCo). Located between the piriform cortex (rostrally), the entorhinal cortex (caudally), and the medial amygdala, it receives direct olfactory input from the OB in layer I and with projections that seem to maintain spatial patterns of the OB (Sosulski et al., 2011). Functionally, it is believed that CoA controls innate odor responses with negative and positive valence (Iurilli and Datta, 2017). In contrast to the basolateral amygdala, the cellular composition of CoA has not been studied extensively, but it is well established that it contains few inhibitory interneurons, most of which are CB or PV-positive (Olucha-Bordonau et al., 2015).

The nLOT represents a unique structure within CoA due to its developmental origin, cellular composition, and connectivity (Santiago and Shammah-Lagnado, 2004). It only comprises about 2,500 neurons and is bi-directionally connected with the OB and PC (Price, 1973; Luskin and Price, 1983). nLOT-lesioned mice display substantial olfactory defects, including decreased detection and discrimination abilities (Vaz et al., 2017). Interestingly, OB input to the nLOT is relatively weak, with more robust input from PC, TT, and basolateral amygdala (Penker et al., 2024). Apart from a low number of VIP-positive interneurons in layer 2, the nLOT has also been shown to contain medium to low numbers of PV, CB, and CR-positive neurons vulnerable to stress-mediated cell atrophy (Vaz et al., 2018).

Neuropeptidergic cells expressing SST, NPY (Real et al., 2009), and VIP (Salamanca et al., 2024) can also be found in CoA and nLOT, mostly in meager numbers. nLOT additionally shows a distinct population of small CCK-positive interneurons (Olucha-Bordonau et al., 2015).

The effects of aging on the entire CoA have not been well described, except for a slight but significant change in volume (Aghamohammadi-Sereshki et al., 2018). In contrast, the volume of nLOT of aged rats showed no noticeable volume reduction. More detailed analysis revealed that aging was associated with a 14% reduction in the total number of nLOT neurons due to cell loss in layers 2 and 3. This included a substantial decline of NPY and VIP positive interneurons with a 55 and 30% reduction, respectively (Figure 2B; Vaz et al., 2016).

The amygdaloid complex also shows substantial atrophy in AD in the early phases of the disease (Poulin et al., 2011). Among these structures, CoA seems to be affected most in terms of senile plaque density, increase in astrocyte number, and volume reduction (Gonzalez-Rodriguez et al., 2023). This study found no reduction in cell numbers but attributed the reduction in volume to neuropil loss and a decrease in synaptic connectivity.

### Lateral entorhinal cortex (LEC)

One main task of the lateral entorhinal cortex (LEC) is to feed nonspatial multisensory information to the hippocampus (Bilash et al., 2023). It has been considered transitional between olfactory allocortices and the isocortex, as its six layers do not precisely match the six layers of the isocortex. The superficial layers (I-III) and deep layers (V and VI) differ strongly in terms of connectivity, with layers II and III receiving cortical inputs and innervating dentate gyrus (DG)/CA3 and CA1/subiculum, respectively, and layers V and VI (deep layers) receiving the output from the hippocampus and sending projections to cortical and subcortical areas as well as the superficial layers of the entorhinal cortex (Wang C. et al., 2023). The LEC receives olfactory information directly from OB and PC, which are both projecting to layer II. It is essential to rapid discrimination of odor identity and intensity (Bitzenhofer et al., 2022) and to be involved in odor-dependent memories and navigation (Li et al., 2017; Radvansky and Dombeck, 2018). With its function in memory formation and retrieval (Pilkiw et al., 2022) and odorant perception (Bitzenhofer et al., 2022), the LEC would be the perfect area to bridge olfactory and cognitive deficits.

The LEC contains a diverse array of GABAergic interneurons that, at least for the superficial layers, have been characterized well in terms of morphology, location, and marker expression (Canto et al., 2008): Layer I contains multipolar cells that express CR and, in a minority of cells, also CB or NPY, and a population of horizontal cells partially expressing VIP. The cell-dense layer II contains many of the principal cells of LEC, pyramidal, and fan cells. Interneurons within layer II are described as multi-polar neurons that can express VIP, CCK, SST, or NPY, horizontal bipolar cells, expressing CR, VIP, CCK, or NPY, as well as PV positive fast-spiking basket and horizontal and vertical chandelier cells (Canto et al., 2008). Layer III also contains chandelier cells and multipolar neurons expressing CCK, SST, or VIP and bipolar cells positive for VIP or CR (Canto et al., 2008). PV-positive interneurons are particularly prominent, comprising approximately half of the interneuron population, especially in layer II, and are essential for synchronizing neural activity and contributing to the oscillatory dynamics of the region (Wouterlood et al., 1995; Miettinen et al., 1996). Furthermore, they can gate information flow in the entorhinal-perirhinal network in a feedforward manner (Willems et al., 2018) and control the output of at least some principal cells to the hippocampus (Nilssen et al., 2018).

The LEC seems to be especially vulnerable to adverse effects in aging and AD (Stranahan and Mattson, 2010) as it shows early changes in aging (Yassa et al., 2010) as well as in AD patients (Igarashi, 2023). Despite substantial changes in function, surprisingly little is known about GABAergic interneurons in both conditions. So far, changes in LEC function in aging have been attributed to changes in intrinsic pyramidal cell excitability (Lin et al., 2022) and expression levels of GABA receptors, as well as GABA synthesizing enzymes, were negligible (Ethiraj et al., 2021).

Substantial alterations in GABAergic neurons have, however, been shown in AD. Significant reductions in the densities of SST and CR interneurons were observed in the LEC of an AD mouse model compared to wild-type (WT) mice (Figure 2A; Klein et al., 2016) while fast-spiking PV-expressing interneurons do not show a significant decline (Ruden et al., 2021).

## Discussion

Olfaction, specifically olfactory dysfunction, has been described to be associated with but precede cognitive decline in aging and AD. Despite intense efforts to define the underlying causes of olfactory decline, our grasp on this issue is still tenuous at best. New evidence suggests that local inhibition might play a more significant part in aging and disease-mediated changes in the brain than previously thought. Therefore, we would also like to promote this idea for research on olfactory dysfunction. We thus summarized knowledge on inhibitory interneurons in different primary olfactory cortical areas to build a basis for examining potential changes. As we have shown the various regions of olfactory information processing are very diverse regarding interneuron content (Figure 1). Furthermore, there are regional differences in the strength of local inhibitory circuits, from the OB consisting predominantly of inhibitory interneurons (Lledo et al., 2008) and the PC which has a, though more simple, similar interneuron makeup to other cortical areas while still heavily relying on feedforward and feedback inhibitory connections for information processing (Franks et al., 2011), to areas with a very low number of inhibitory interneurons like CoA (Olucha-Bordonau et al., 2015).

Changes in interneuron function, like GABA expression and oscillatory activity, as well as changes in interneuron number in AD, have been shown in multiple brain areas (Xu et al., 2020). So far, the olfactory areas included in this line of research show similar interneuronal changes as the hippocampus and cortex, with PV—and CR-positive neuron decline only detectable in some mouse models and brain areas. In contrast, SST-positive neuron numbers have been shown to decrease invariably.

Research on “olfactory aging” has been sparse, leaving interneuronal changes poorly defined. The existing literature, however, already reflects that a decrease in interneuron numbers is far less pronounced than in AD. Though a lot more research is required, our review thus shows that changes in inhibitory local networks mirror the current view derived from wider brain areas that neuronal cell loss was found predominantly in pathological aging, such as AD, while normal aging is accompanied by dendritic, synaptic, and axonal degeneration with nearly no cell loss (Schliebs and Arendt, 2011). This leads to the question of whether these processes are fundamentally different or just occurrences on a different time scale. Would a better understanding of olfactory changes enable us to create a more detailed battery of olfactory tests to detect neurodegenerative diseases, or is this delineation impossible?

Our review deals with the loss of olfactory interneurons as a proxy and the most apparent sign of reduced inhibitory drive in the different olfactory brain areas. Other detectable signs are, among others, a reduction of iPSCs in patch clamp recordings, a reduction of synaptic connections in local circuits, and an increase in the activity of principal neurons (Meftah and Gan, 2023). A decrease in GABA release can cause these changes but they might, at least partially, stem from a reduction in GABA receptor-mediated signaling at the postsynapse (Sakimoto et al., 2021). GABA_A_ receptors seem to be mainly involved in Aβ-associated pathology as the expression of several subunits of GABA_A_ receptors is changed in AD patients (Kwakowsky et al., 2018), and pre-treatment of rat cortical cell cultures with a GABA_A_ antagonist was able to inhibit Aβ-induced neuronal apoptosis (Lee et al., 2005). In this respect, it is interesting that expression levels of GABA_A_ receptors are very different between different olfactory areas, with, e.g., the AON showing little GABA_A_ receptor density and several of the olfactory brain areas showing substantial differences in receptor density in other layers (Lothmann et al., 2021). This raises the possibility that different olfactory areas might show similar numbers of senile plaques but are not equally vulnerable to neuronal deterioration due to Aβ deposition.

Apart from differences in vulnerability, an increase in olfactory interneuron-related research might further be essential to understand olfactory dysfunction as interneurons are involved in the function of local networks in numerous ways.

### Aberrant excitation

We have mentioned previously that a change in local inhibitory drive can lead to aberrant excitation of principal cells in any brain area. This increase in excitation on a single cell level has not only been shown in the hippocampus (Targa Dias Anastacio et al., 2022) but also in a slice preparation of the OB (Li et al., 2019) and in OB, PC (Wesson et al., 2011b) and the lateral entorhinal cortex of anesthetized mice in an AD model (Xu M. et al., 2015). An increase in excitatory activity and a decrease in inhibitory drive might explain the effects on discrimination abilities by increasing sensory fields and affect detection due to the reduction in signal-to-noise ratio.

### Oscillatory activity

Hyperexcitatory activity can also be seen on the network level in the form of local field potentials (LFP). Olfactory LFP activity is commonly organized into the theta or the “respiratory” band (2–12 Hz), the beta band (18–30 Hz), and the gamma band (30–100 Hz), with each of those bands representing unique aspects of odor perception (Kay et al., 2009). It was shown that OB and PC of transgenic mice of an AD model exhibit a detectable shift in LFP power already at 3–4 months of age preceding the reduction in olfactory performance (Wesson et al., 2011b). A couple of publications were able to confirm changes in LFP in the OB of AD models (Li et al., 2019; Chen et al., 2021). As at least gamma frequency bands of the LFP stem predominantly from the interplay of granule cells with mitral cells in the EPL, the finding corresponds well with findings of the impairment of dendrodendritic inhibition between these two cell types in an AD model (Li et al., 2019; Chen et al., 2021).

Changes in gamma band oscillations from the OB have also been shown in aging mice (Ahnaou et al., 2020) presumed to stem from a reduction in PV neuron function in the EPL. Gamma band oscillations have been described to be specifically crucial for olfactory discrimination (Nusser et al., 2001) and olfactory processing (Martin and Ravel, 2014) and, therefore, may account for the decline in olfactory performance.

### Olfactory bulb plasticity

The olfactory bulb is one of the few brain areas that exhibit lifelong regeneration. Neuronal progenitor cells from the rostral migratory stream are integrated into the glomerular and granule cell layer and differentiate into mostly calretinin-positive neurons in an input-dependent manner (Kim and Choe, 2020). As we have mentioned above, a decrease in neuro-regeneration in the OB is supposed to be one of the main underlying factors of olfactory dysfunction in aging and AD (Dibattista et al., 2020). Proliferation in the subventricular zone and rostral migratory stream declines with age and in AD. That this dwindling number of migrating neuroblasts is causing changes in the OB circuit is, however, far from clear and seems to stem mainly from notions gained from HC neurogenesis (Choi and Tanzi, 2023). Changes in CR-positive neurons have not been shown for the aging OB (Mobley et al., 2013) while in an AD mouse model, the OB seems to exhibit a smaller amount of adult-born neurons in the glomerular layer that does, however, not significantly affect the numbers of CR-positive neurons (La Rosa-Prieto et al., 2016). More research is required to clarify this critical issue as newly integrated neurons have been shown to affect gamma-band oscillations in the OB and affect olfactory discrimination and odor memory (Sakamoto et al., 2014).

### Interneurons as targets for top-down cholinergic input

A hallmark of brain dysfunction in aging and neurodegenerative diseases is reduced cholinergic input from the basal forebrain to different brain areas. This reduction is well described in AD as well as in aging (Schliebs and Arendt, 2006; Sultzer, 2018; Chen et al., 2022). As these neuromodulatory inputs also target local inhibitory interneurons, they can strongly affect the function of inhibitory interneurons (Picciotto et al., 2012). The role of acetylcholine (ACh) in the olfactory system has yet to be clarified. For the olfactory bulb, it has been shown that cholinergic input increases activity in output neurons indiscriminately (Rothermel et al., 2013; Boehm et al., 2020). Therefore, a decrease in input would be expected to dampen OB output. In the piriform cortex, ACh has been shown to inhibit recurrent excitatory activity, specifically (Hasselmo and Bower, 1992) Therefore, reducing ACh would mean increased recurrent excitation and a more robust gating of olfactory information at this processing level. As the cholinergic system innervates all other levels of olfactory processing, the net effect of reduced cholinergic modulation is hard to predict. Still, it is feasible that cholinergic decline is also an underlying factor for olfactory dysfunction.

## Conclusion

Inhibitory interneurons and their function in regulating local circuit functions are of great importance for cognitive function in the hippocampus and, as we have shown in this review, for olfactory function. Olfactory information processing relies heavily on local inhibitory effects, especially in the early stages like the OB (Burton, 2017). This inhibition might play a large part in the manifestation of olfactory behavioral symptoms in AD and aging.

Our comparison of reports on interneuron changes in aging and AD shows that these alterations are different between the two conditions but similar to those shown for cortical and hippocampal regions. In this respect, neuronal populations expressing SST and PV seem most vulnerable to AD pathological aging. As these neurons are not expressed in higher numbers in the olfactory system, these changes cannot explain the early and robust manifestation of olfactory dysfunction.

Previous reports have shown that hyperexcitability in various olfactory areas like OB, PC (Wesson et al., 2011a), and LEC (Xu W. et al., 2015) precedes behavioral and hippocampal dysfunction and a detectable decrease in cell numbers in an AD mouse model. Therefore, one might speculate that the earliest symptoms are GABAergic dysfunctions on a synaptic level, similar to what has been shown for aging (Rozycka and Liguz-Lecznar, 2017). The increase in hyperexcitability on both peripheral and central olfactory circuits also raises the possibility that not one olfactory area is responsible for olfactory deficits but that a concerted effort of all olfactory regions is required to enable discrimination, detection, and recognition. This would explain why experimental disturbances on every level of the olfactory processing and many neurodegenerative diseases create similar deficits. This theory of “wholistic” perception, under robust inhibitory control, might help us to further our understanding of the olfactory system as a warning system for changes in brain function.
